# The Overlooked Sad Face Expression: Understanding the Omega Sign in Botulinum Toxin Treatment

**DOI:** 10.1111/jocd.70200

**Published:** 2025-06-04

**Authors:** Cristina Muñoz‐Gonzalez, Juan Martin Zarate, Nabil Fakih‐Gomez

**Affiliations:** ^1^ Department of Facial Plastic and Cranio‐Maxillo‐Facial Surgery Fakih Hospital Khaizaran Lebanon; ^2^ Department of Surgery University of Salamanca Salamanca Spain

**Keywords:** botulinum toxin, corrugator supercilii muscle, omega pattern, sad expression, sad face

## Abstract

**Introduction:**

Botulinum toxin injections are a popular cosmetic treatment for smoothing wrinkles. Recent trends emphasize not just wrinkle reduction but also the correction of facial expressions that convey emotions. Among these, the sad face expression, associated with the omega pattern, is often overlooked despite its significant role in emotional expression.

**Methods:**

This study explored the anatomy and muscle activity related to the sad face expression, focusing on the occipitofrontalis, corrugator supercilii, depressor supercilii, procerus, and orbicularis oculi muscles. A new classification system was developed to categorize this expression based on patient control and muscle strength.

**Results:**

The study found that the omega pattern is produced by the interaction of specific facial muscles, with three types of patient muscle control identified: automatic, controllable, and uncontrollable gestures. The visibility of the sad face expression varies among individuals due to differences in muscle strength and control.

**Conclusion:**

Incorporating the assessment of the sad face expression into pretreatment evaluations can enhance the effectiveness of botulinum toxin treatments by addressing both aesthetic and emotional aspects of facial expressions.

**Level of Evidence:**

V.

## 1 Introduction

Botulinum toxin injections have become the most popular minimally invasive cosmetic procedure worldwide, primarily recognized for their ability to smooth wrinkles. However, the focus has gradually shifted from merely addressing wrinkles to correcting facial expressions, which play a crucial role in conveying emotions. The relationship between botulinum toxin and emotional expression is gaining increasing attention, as studies suggest that altering facial expressions through these injections can influence emotional well‐being. Emotions such as anger, surprise, happiness, and sadness are often expressed through five distinct glabellar contraction patterns, each leading to different wrinkle formations [1]. These patterns, identified as “U,” “V,” “Omega,” “Inverted Omega,” and “Converging Arrows,” have been widely recognized in the evaluation of facial wrinkles in aesthetic medicine [2, 3].

Despite the importance of these patterns, the sad face expression, associated with the omega pattern, has been largely overlooked in clinical assessments, even though it plays a significant role in emotional expression. In botulinum toxin treatments, assessments typically focus on the surprise, happy, and angry gestures, often neglecting the expression of sadness. Evaluating patients for botulinum toxin injections usually involves assessing the contraction patterns of facial muscles by asking them to frown (glabellar lines), elevate the brows (forehead lines), and smile (crow's feet). However, variations in these frowning manifestations have been noted. For instance, in some individuals when frowning, the brow moves downwards, creating an angry face, while in others, the brow head moves upwards, depicting a sad face. These variations highlight the complexity of facial expressions, which are not merely isolated muscle movements but also reflections of underlying emotions. Notably, there is a lack of substantial literary evidence on assessing the sad face expression, which this article aims to address by helping clinicians identify this expression and include it in preoperative assessments for optimal treatment of facial wrinkles.

The omega pattern is characterized by the approximation and elevation of the glabella to form the shape of the Greek letter omega [1, 4]. This pattern is closely linked to feelings of sadness and melancholy, as first described by Darwin, and is thought to be triggered by specific emotional states, particularly during stress or agitation [5, 6]. Although its incidence is reported to be relatively low, authors argue that nearly everyone is capable of producing this expression. The omega pattern may not be solely determined by individual contraction patterns but rather serves as a reflection of an emotional state, similar to how raising the brows might be associated with surprise. Variations in the anatomy and strength of the muscles involved can lead to differences in the visibility of the omega pattern across individuals.

This study examines the sad face expression, correlating it with the responsible musculature and identifying the causes behind this gesture. Recognizing the importance of this expression is crucial when administering botulinum toxin injections. By integrating the sad face expression, particularly the omega pattern, into preoperative assessments, clinicians can better address the emotional and psychological aspects of facial aesthetics, ultimately enhancing patient satisfaction and well‐being [1, 7].

## 2 Materials and Methods

### 2.1 Anatomical Consideration

Understanding the sad face expression requires an in‐depth knowledge of the anatomy of the mimetic muscles responsible for facial expressions, which play a critical role in nonverbal communication.

- Occipitofrontalis muscle: Often referred to as a single muscle, the occipitofrontalis consists of two independent parts: the frontalis (anterior) and occipitalis (posterior) bellies [8]. This muscle attaches to the eyebrow, with its vertical fibers interdigitating with those of the procerus, corrugator supercilii muscle (CSM), depressor supercilii (DSM), and orbicularis oculi muscle (OOM) [8]. When the occipitofrontalis contracts, it elevates the eyebrows and forms horizontal wrinkles across the forehead [9]. The muscle presents in four distinct configuration patterns, each leading to different forehead wrinkling patterns:
  Type I: Full‐form bellies that meet at the midline, creating straight lines across the forehead.
  Type II: V‐shaped bellies separated by a V‐shaped aponeurotic galea, forming gull‐wing‐shaped lines.
  Type III: Central form bellies that join above the medial half of the orbital rims, creating short lines in the middle of the forehead.
  Type IV: Lateral form bellies that create two short lateral columns of lines, leaving the middle area smooth [9].
  The occipitofrontalis muscle is responsible for elevating the eyebrows.
- CSM: Located beneath the frontalis and OOM, the CSM is part of the deep forehead muscle layer. It originates approximately 16 mm above the horizontal intercanthal plane and 4–14 mm from the midline, extending at a 30° angle to insert into the dermis roughly 30 mm above the same plane [10, 11, 12, 13]. Despite having two bellies, the CSM functions as a single muscle: the transverse (lateral) and oblique (medial). The transverse head runs laterally, blending with the OOM and frontalis muscles. In contrast, the oblique head extends parallel to the DSM and attaches to the medial brow. It can be either narrow, merging with the frontalis in the medial third, or broad, covering more area and producing stronger eyebrow contractions. The two heads often merge, making them difficult to distinguish [14, 15, 16]. The CSM is innervated by the frontal and angular nerves. The transverse head primarily receives innervation from the frontal nerve, whereas the oblique head is supplied by the angular nerve [17, 18]. Traditionally recognized as a brow depressor, the CSM collaborates with the medial OOM to create vertical glabellar furrows, commonly associated with expressions of anger or concentration [9, 11, 19, 20, 21, 22]. However, newer studies suggest that the CSM's structural features allow it to elevate and medialize the medial brow, depending on its interaction with the frontalis and OOM [23, 24, 25].
- DSM: once considered part of the CSM, is now recognized as an independent, fan‐shaped muscle. It originates from the frontal process of the maxilla, approximately 1 cm above the medial canthal ligament, and inserts into the skin beneath the medial eyebrow. This anatomical distinction, confirmed by detailed studies, highlights the DSM's unique role in facial expression, particularly in depressing the medial brow and contributing to the formation of vertical rhytids [26, 27]. Botulinum toxin injections targeting the DSM can elevate the medial brow, making it a critical focus in aesthetic treatments [25, 28, 29].
- Procerus muscle: The procerus muscle is pyramid‐shaped, originating from both the superficial and deep layers at the midline of the nasal bone and cartilage. It inserts into the skin of the lower forehead between the eyebrows and into the frontalis muscle belly. When contracted, its vertical fibers depress the medial eyebrow angle, forming a transverse fold across the nasal root and creating horizontal rhytids [9].
- OOM: Located just beneath the skin of the eyelids, it plays a vital role in eyelid movement. Extending from the medial to the lateral canthal regions, it contributes significantly to both the structural integrity and functionality of the eyelids. As a sphincter‐like muscle, it encircles the upper and lower eyelids in a concentric pattern. Its primary function is to close the eyelids, which is crucial for protecting the eyes and facilitating tear drainage. The muscle is divided into two main sections: the orbital and palpebral, each further subdivided to perform specialized tasks. Notably, the orbital portion of the muscle, in conjunction with the CSM and DSM, acts as a depressor of the lateral, central, and medial parts of the eyebrow, influencing various facial expressions [30].

All patients included in this study provided written informed consent for the use of their medical records and data extraction for research purposes. Additionally, all participants granted permission for the publication of their photographs.

## 3 Results

### 3.1 Muscle Activity in Key Facial Expressions

**FIGURE 1:**
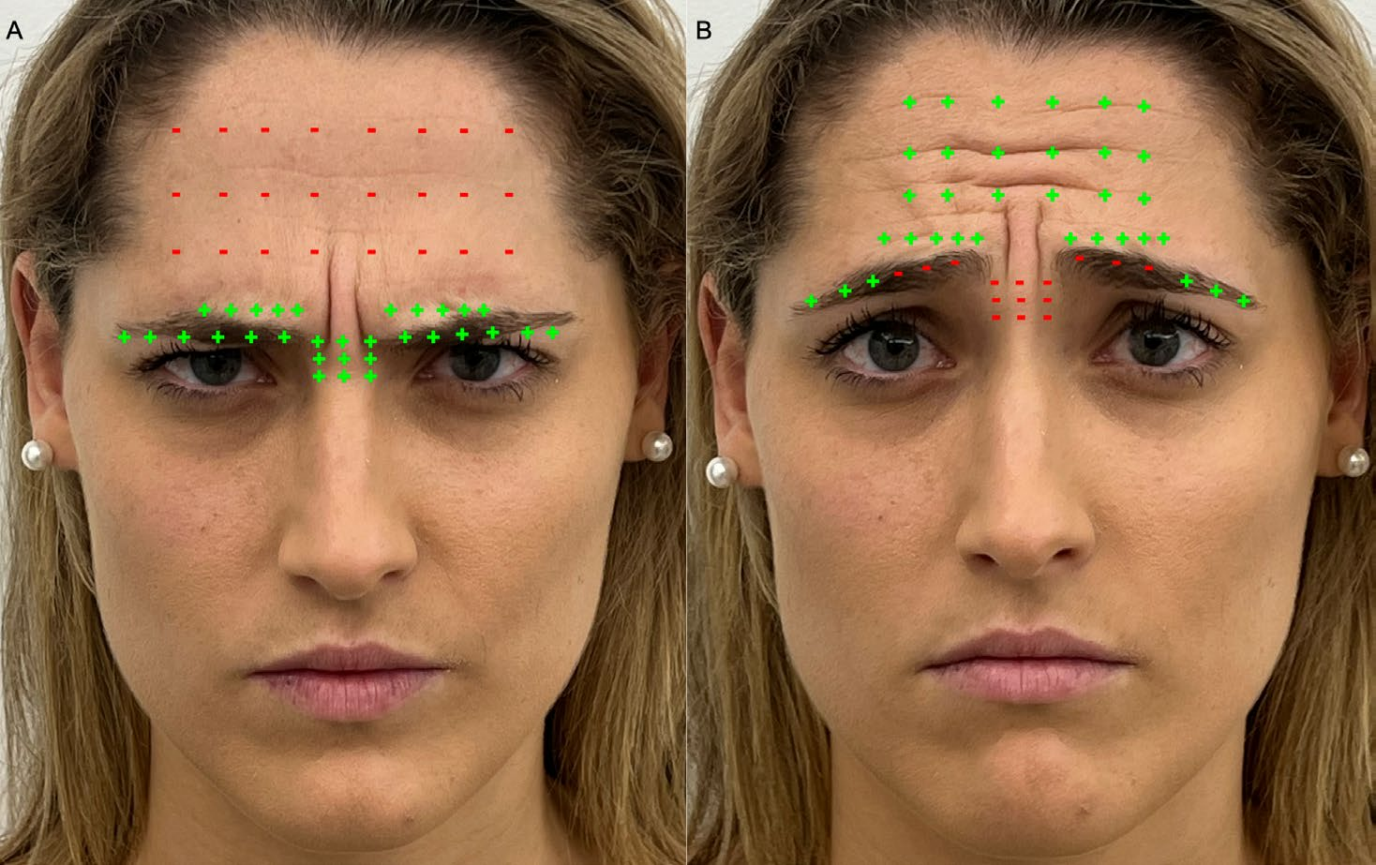
Muscle activation patterns in angry face and sad face expressions, illustrated in a 26‐year‐old female subject. Active muscles are shown in green, and inactive muscles in red. (A) Angry face gesture: Shows activation of the DSM, CSM, procerus, and OOM. These muscles work together to pull the brows medially and downward, creating vertical glabellar lines (J lines, central lines). The DSM and procerus primarily act as depressors, while the CSM medializes the brow and the OOM contracts around the eyes, all while the frontalis muscle remains relaxed. (B) Sad face gesture: Involves a combination of the CSM (transverse and oblique portions) and the frontalis muscle, forming an “omega sign” on the forehead. This expression is characterized by elevation of the medial brow and depression of the lateral brow, with a distinct “Ω” shape. For the sad face to be prominent, the DSM, medial OOM, and procerus must remain inactive, allowing the CSM and frontalis to elevate the medial brow.

Facial expressions like surprise, sadness, and anger result from the coordinated activity of various facial muscles. A deep understanding of these muscle interactions is essential for both aesthetic and therapeutic applications:

- Surprise gesture: The surprise expression is primarily driven by the contraction of the frontalis muscle, which elevates the eyebrows and creates horizontal forehead wrinkles. This action is relatively simple, involving only the frontalis muscle, which is the main elevator of the eyebrows. In this gesture, all other involved muscles remain relaxed, allowing the expression to emerge fully.
- Angry face gesture: The angry expression involves the activation of the DSM, CSM, procerus, and OOM. These muscles collectively pull the eyebrows downward and medially, forming vertical glabellar furrows (J lines, central lines). The DSM acts as a primary depressor in conjunction with the procerus; the CSM medializes the brow while the OOM enhances the frown by contracting around the eyes. It is important to note that the transverse portion of the CSM, procerus, and DSM are interconnected through their fibers with the OOM, working together to depress the brows while the frontalis muscle remains relaxed.
- Sad face gesture: The sad face expression activates a combination of the frontalis and CSM (transverse and oblique portions), forming the distinctive omega sign on the forehead. This pattern emerges when the CSM and frontalis muscles work together to elevate the medial brow while depressing the lateral brow. For this expression to be clearly visible, the DSM and procerus must remain relaxed, allowing the other muscles to perform their roles. The interplay between these muscle groups—some functioning as elevators and others as depressors—is what creates the characteristic sad face expression. The omega sign itself, an “Ω” shape on the forehead, results from the coordinated movement of these muscle groups. The frontalis and CSM elevate the medial brow, while the lateral portion of the OOM contributes to the downward pull of the lateral brow. At the same time, the DSM, the medial part of the OOM, and the procerus remain relaxed, accentuating the curvature of the omega sign. The prominence and visibility of this expression depend on the balance of these muscle forces, reflecting the underlying anatomical and muscular dynamics (Figure 1).

### 3.2 Classification of Sad Face Gesture

Sad face gestures can be classified into three distinct types based on the patient's ability to control the expression and the underlying muscle strength (Video S1):

- Automatic gesture (Class 1): The sad face gesture appears automatically, often seen in patients with a hypokinetic DSM and procerus. This weakness allows the remaining elevator muscles to dominate, creating the movement spontaneously. Patients in this category can easily produce the gesture, even when attempting an angry face expression.
- Controllable gesture (Class 2): It involves a balanced muscle force, allowing patients to control and consciously produce the sad face gesture. This category is advantageous for actors or individuals who can be coached to create this expression on command. In these cases, simply instructing the patient to make a sad face or an angry face often results in the desired expressions.
- Uncontrollable or weak gesture (Class 3): It is characterized by an inability to fully control the gesture, typically due to weaker central frontalis strength, and/or the CSM with the oblique portion potentially contributing to the weakness, combined with an excessively hyperkinetic DSM and procerus muscle. Consequently, the sad face may appear faint, incomplete, or suggest an inability to fully produce the expression. To identify this gesture, a specific maneuver can be used: First, the patient is asked to raise their eyebrows. Then, the practitioner places their thumb on the central glabellar region and applies upward pressure to block it. While maintaining this block, the patient is instructed to frown. Afterward, they are asked to relax the central glabellar area while the pressure remains applied. Once the practitioner releases the block, the sad face expression usually becomes noticeable (Video S2). This technique often reveals a more subtle version of the gesture.

## 4 Discussion

The intricate relationship between facial muscle activity and emotional expression has been a central focus in both medical and psychological research for many years. Hyperkinetic activity in mimetic muscles, such as the procerus, DSM, and CSM, plays a crucial role in the formation of facial wrinkles, which serve as indicators of various emotional states. Horizontal frown lines in the glabellar region are primarily caused by the procerus and DSM, while vertical lines are predominantly attributed to the CSM, with some contribution from the OOM [19].

Patient evaluations have revealed that the CSM and lower frontalis muscles can create a sinusoidal contraction pattern at the forehead's center, contributing to the sad face expression. Traditionally, the CSM is recognized for generating vertical furrows associated with emotions of suffering; however, it also plays a role in the upward movement of the brow. This movement cannot be solely attributed to the CSM, indicating a more complex interaction among facial muscles. Specifically, the CSM works synergistically with the frontalis muscle medially and with the OOM laterally, while acting antagonistically against the procerus and OOM at the brow [23, 24, 25]. This complex muscle dynamics highlight the intricacy of brow movement and expression.

It is noteworthy that the relationship between the omega sign and Veraguth's sign (lateral wrinkles accompanying the omega shape, causing brow depression) has been recognized historically in connection with melancholy. Darwin first described this phenomenon in 1872, linking it to the contraction of the corrugator muscle [5]. This observation was later validated by findings from an electromyography study conducted in 1985, where patients who imagined scenarios evoking fear or sadness exhibited the omega sign, with the corrugator muscle being the primary one involved. These findings confirm and extend earlier historical observations [6].

Behavioral research has long identified six universal facial expressions: disgust, anger, sadness, happiness, surprise, and fear [31]. Each expression correlates with distinct muscle activities and emotional states (Video S3). Despite its universal significance, the sad face expression—marked by the omega sign—has frequently been overlooked in aesthetic medicine, even though it is well‐recognized in other fields such as behavioral science. This gesture involves complex muscle interactions, including the procerus and CSM. It is important to note that the sad face and fear expressions are similar, both characterized by the omega sign, but they differ in the lower third of the face [32].

**FIGURE 2:**
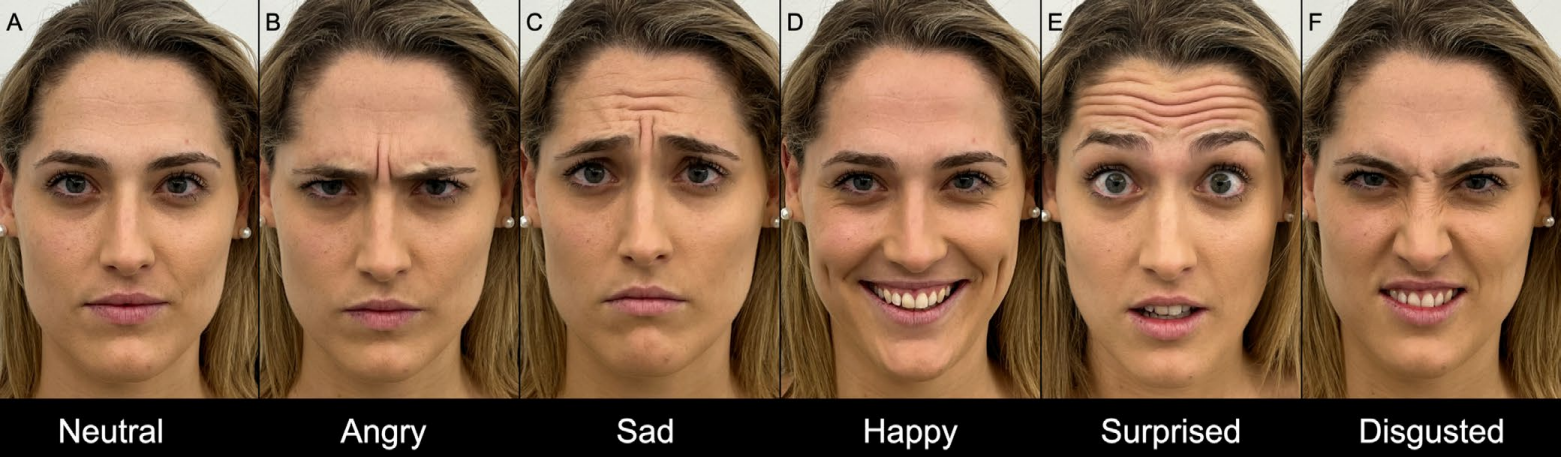
Illustration of the recommended pretreatment assessments with photographic documentation, featuring frontal view photographs that capture a full range of facial expressions in a 26‐year‐old female subject: (A) Neutral, (B) Angry, (C) Sad, (D) Happy, (E) Surprised, and (F) Disgusted.

To improve the precision of botulinum toxin treatments in the upper third of the face, the authors recommended that pretreatment assessments and photographic documentation include a series of frontal view photographs capturing a full range of facial expressions: neutral, angry, surprised, happy, disgusted, and sad (Figure 2). This comprehensive approach enables a detailed evaluation of muscle dynamics and emotional expressions, resulting in more accurate and effective treatment outcomes.

Findings indicate that while all individuals have the musculature necessary to produce universal facial expressions, such as surprise or happiness, the same holds true for the sad face in facial mimics. The visibility and intensity of these expressions vary depending on muscle strength and control, which is particularly evident in the sad face. As Vanessa Van Edwards notes, sadness is the hardest emotion to fake, and it is less frequently practiced or observed in daily life, making it more challenging to identify and replicate [32, 33]. This might explain why previous research incorrectly classified it as a mere contraction pattern rather than a universal emotion [1, 2, 3, 4]. Previous studies, such as those by Almeida et al. and Kamat et al., have reported low percentages (10.2%–15%) of individuals displaying the sad face expression, likely due to this misunderstanding [1, 2]. Moreover, a recent study by Cotofana et al. reinforces the authors' conclusions, showing that in the cohort and Magnetic Resonance Imaging parameters examined, there appears to be minimal correlation between glabellar contraction patterns and the underlying muscle anatomy in the glabella. Thus, designing neuromodulator treatment plans based on these contraction patterns may offer limited clinical benefit [34].

**FIGURE 3:**
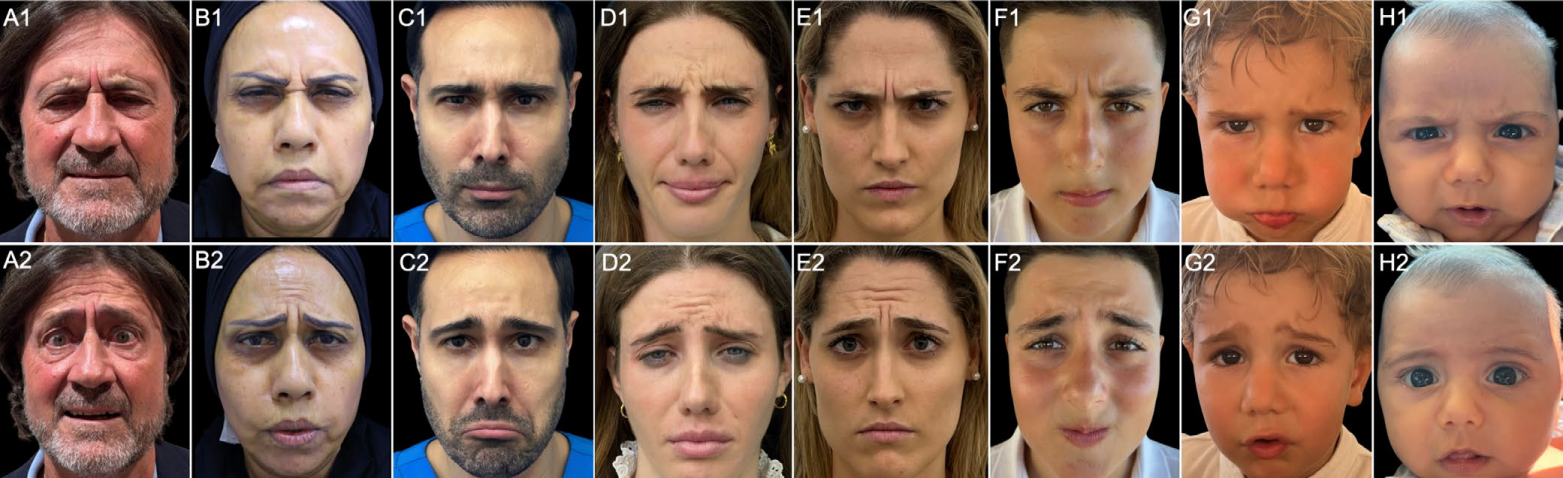
Observations across a diverse patient population, ranging from 3 months to 65 years, revealed variability in the expression of angry and sad gestures across different age groups, with all participants being capable of performing both expressions. The numbering denotes the specific action being performed: “1” represents brow furrowing for the angry expression, while “2” indicates the sad facial expression. The letter labels correspond to patient age groups in each decade: (A) male in his sixties, (B) female in her fifties, (C) male in his forties, (D) female in her thirties, (E) female in her twenties, (F) male in his teens, (G) male aged 3 years, and (H) male aged 3 months.

Author's observation across a diverse patient population, ranging from 3 months to 65 years, showed that nearly all could produce the angry face, but the sad face expression exhibited significant variability (Figure 3). This observation underscores the need for personalized assessments in clinical and aesthetic practices. The similarity between the fear and sad face expressions was also observed in a 3‐month‐old subject (Figure 3: H1, H2). Inducing sadness proved difficult, so it was opted to evoke fear, which successfully triggered the omega sign.

These insights, combined with our previous publication on the CSM's role, shed light on why the omega sign becomes more pronounced when using a modified technique [25]. By selectively blocking the DSM while leaving the oblique portion of the CSM unaffected during botulinum toxin treatment, we produced an undesirable omega sign (automatic Class 1) effect when frowning [25]. Studies have shown that patients exhibiting the omega sign may require higher doses targeting the CSM while avoiding treatment of the procerus [4], a finding supported by the author's research as well [25]. Additionally, Kim et al. suggest that this glabellar contraction pattern is often linked to a hypokinetic procerus muscle, which may sometimes be divided into two lateral portions [3].

This comprehensive understanding of muscle function and the sad face gesture highlights the need for a more refined technique, one that emphasizes both anatomical and emotional considerations in toxin administration. The authors are actively developing a novel injection technique based on these findings to achieve natural facial expressions following botulinum toxin treatment. This approach aims to prevent the formation of omega lines while maintaining a higher eyebrow position compared to traditional methods. The results of this research are currently being prepared for publication.

## 5 Conclusion

This study highlights the importance of incorporating the sad face gesture (omega sign) in botulinum toxin assessments. By understanding the muscle dynamics of this expression, clinicians can improve treatment outcomes, balancing cosmetic and emotional considerations. Future research will focus on refining injection techniques to prevent omega lines, ensure natural expressions, and maintain ideal eyebrow positioning.

## Author Contributions

C.M.‐G., J.M.Z., and N.F.‐G. have made substantial contributions to the conception and design of the study, as well as the acquisition and interpretation of data. C.M.‐G. and N.F.‐G. were involved in drafting the manuscript and critically revising it for important intellectual content. All authors have given their final approval of the version to be published. Furthermore, C.M.‐G., J.M.Z., and N.F.‐G. agree to be accountable for all aspects of the work, ensuring that any questions related to the accuracy or integrity of any part of the study are appropriately investigated and resolved.

## Disclosure

Animal and human rights: All treatments were performed in adherence with the Declaration of Helsinki and in accordance with the standards of good clinical care following local guidelines and regulations. This article does not contain any studies with animals performed by any of the authors.

Data Policy: For this type of study, we do not have data to deposit in a public repository.

Consent to Publish: All participants have provided consent for the publication of their photographs.

## Consent

All patients included in this study provided written informed consent for accessing their patient charts and extracting their data for the purposes of this study. No charts were accessed if patients declined their participation in this study.

## Conflicts of Interest

The authors N.F.G., C.M.G., and J.M.Z. are consultants for Merz Aesthetics (Frankfurt, Germany).

## Supporting information


**Video S1.** This video demonstrates the three types of sad face gestures, classified based on the patient’s ability to control the expression and the underlying muscle strength. First, we observe a male patient displaying an automatic gesture. Next, a female patient is shown with a controllable gesture. Lastly, an uncontrollable or weak gesture in another female patient with a subtle omega sign is presented.

**Video S2.** This video demonstrates the maneuver to identify the sad face gesture in a female patient. The patient is first instructed to raise their eyebrows, followed by the practitioner applying upward pressure on the central glabellar region to block it. While maintaining the block, the patient frowns. After relaxing the glabellar area, the sad face expression typically becomes more apparent once the block is released, often revealing a more subtle version of the gesture.

**Video S3.** This video highlights the universal facial expressions identified by behavioral research that should be observed during the preassessment of patients: relaxed (neutral), anger, surprise, sadness, happiness, and disgust.

## Data Availability

The data that support the findings of this study are available from the corresponding author upon reasonable request.

## References

[1] A. R. de Almeida , E. R. da Costa Marques , R. Banegas , and B. V. Kadunc , “Glabellar Contraction Patterns: A Tool to Optimize Botulinum Toxin Treatment,” Dermatologic Surgery 38, no. 9 (2012): 1506–1515, 10.1111/j.1524-4725.2012.02505.x. 22804914

[2] A. Kamat and T. Quadros , “An Observational Study on Glabellar Wrinkle Patterns in Indians,” Indian Journal of Dermatology, Venereology and Leprology 85, no. 2 (2019): 182–189, 10.4103/ijdvl.IJDVL_211_17. 29620040

[3] S. B. Kim , H. M. Kim , H. Ahn , et al., “Anatomical Injection Guidelines for Glabellar Frown Lines Based on Ultrasonographic Evaluation,” Toxins (Basel) 14, no. 1 (2021): 17, 10.3390/toxins14010017. 35050994 PMC8778322

[4] A. Borba , S. Matayoshi , and M. Rodrigues , “Avoiding Complications on the Upper Face Treatment With Botulinum Toxin: A Practical Guide,” Aesthetic Plastic Surgery 46, no. 1 (2022): 385–394, 10.1007/s00266-021-02483-1. 34341857 PMC8328485

[5] C. Darwin , The Expression of the Emotions in Man and Animals (John Murray, 1872), 374.

[6] J. F. Greden , N. Genero , and H. L. Price , “Agitation‐Increased Electromyogram Activity in the Corrugator Muscle Region: A Possible Explanation of the “Omega Sign”?,” American Journal of Psychiatry 142, no. 3 (1985): 348–351, 10.1176/ajp.142.3.348. 3970275

[7] H. Jiang , J. Zhou , and S. Chen , “Different Glabellar Contraction Patterns in Chinese and Efficacy of Botulinum Toxin Type A for Treating Glabellar Lines: A Pilot Study,” Dermatologic Surgery 43, no. 5 (2017): 692–697, 10.1097/DSS.0000000000001045. 28244900

[8] B. R. Costin , T. P. Plesec , N. Sakolsatayadorn , T. J. Rubinstein , J. M. McBride , and J. D. Perry , “Anatomy and Histology of the Frontalis Muscle,” Ophthalmic Plastic and Reconstructive Surgery 31, no. 1 (2015): 66–72, 10.1097/IOP.0000000000000244. 25417794

[9] A. D'Souza and C. L. Ng , “Applied Anatomy for Botulinum Toxin Injection in Cosmetic Interventions,” Current Otorhinolaryngology Reports 8 (2020): 336–343, 10.1007/s40136-020-00308-4.

[10] J. E. Janis , A. Ghavami , J. A. Lemmon , J. E. Leedy , and B. Guyuron , “Anatomy of the Corrugator Supercilii Muscle: Part I. Corrugator Topography,” Plastic and Reconstructive Surgery 120, no. 6 (2007): 1647–1653, 10.1097/01.prs.0000282725.61640.e1. 18040200

[11] K. Hwang , J. H. Lee , and H. J. Lim , “Anatomy of the Corrugator Muscle,” Journal of Craniofacial Surgery 28, no. 2 (2017): 524–527, 10.1097/SCS.0000000000003304. 28005653

[12] J. L. Walden , C. C. Brown , A. J. Klapper , C. T. Chia , and S. J. Aston , “An Anatomical Comparison of Transpalpebral, Endoscopic, and Coronal Approaches to Demonstrate Exposure and Extent of Brow Depressor Muscle Resection,” Plastic and Reconstructive Surgery 116, no. 5 (2005): 1479–1487, 10.1097/01.prs.0000182649.14511.52. 16217498

[13] D. M. Knize , “An Anatomically Based Study of the Mechanism of Eyebrow Ptosis,” Plastic and Reconstructive Surgery 97, no. 7 (1996): 1321–1333, 10.1097/00006534-199606000-00001. 8643714

[14] K. H. Yi , J. H. Lee , H. W. Hu , and H. J. Kim , “Anatomical Proposal for Botulinum Neurotoxin Injection for Glabellar Frown Lines,” Toxins (Basel) 14, no. 4 (2022): 268, 10.3390/toxins14040268. 35448877 PMC9032255

[15] H. M. Yang and H. J. Kim , “Anatomical Study of the Corrugator Supercilii Muscle and Its Clinical Implication With Botulinum Toxin A Injection,” Surgical and Radiologic Anatomy 35, no. 9 (2013): 817–821, 10.1007/s00276-013-1174-5. 23897537

[16] J. M. Wieder and R. L. Moy , “Understanding Botulinum Toxin. Surgical Anatomy of the Frown, Forehead, and Periocular Region,” Dermatologic Surgery 24, no. 11 (1998): 1172–1174. 9834734

[17] D. M. Caminer , M. I. Newman , and J. B. Boyd , “Angular Nerve: New Insights on Innervation of the Corrugator Supercilii and Procerus Muscles,” Journal of Plastic, Reconstructive & Aesthetic Surgery 59, no. 4 (2006): 366–372, 10.1016/j.bjps.2005.09.011. 16756251

[18] B. Eshraghi , H. Ghadimi , M. Nikdel , and F. Hajizadeh , “Synkinesis Between Orbicularis Oculi and Procerus Muscles: Video Presentation of an Unusual Type of Aberrant Innervation After Cosmetic Rhinoplasty,” Aesthetic Plastic Surgery 43, no. 1 (2019): 98–101, 10.1007/s00266-018-1255-2. 30327854

[19] A. V. Benedetto and J. G. Lahti , “Measurement of the Anatomic Position of the Corrugator Supercilii,” Dermatologic Surgery 31, no. 8 (2005): 923–927, 10.1097/00042728-200508000-00006. 16042937

[20] A. C. Abramo , “Anatomy of the Forehead Muscles: The Basis for the Videoendoscopic Approach in Forehead Rhytidoplasty,” Plastic and Reconstructive Surgery 95, no. 7 (1995): 1170–1177, 10.1097/00006534-199506000-00005. 7761503

[21] J. E. Janis , A. Ghavami , J. A. Lemmon , J. E. Leedy , and B. Guyuron , “The Anatomy of the Corrugator Supercilii Muscle: Part II. Supraorbital Nerve Branching Patterns,” Plastic and Reconstructive Surgery 121, no. 1 (2008): 233–240, 10.1097/01.prs.0000299260.04932.38. 18176226

[22] M. S. Hur , S. Lee , H. S. Jung , and R. A. Schneider , “Anatomical Connections Among the Depressor Supercilii, Levator Labii Superioris Alaeque Nasi, and Inferior Fibers of Orbicularis Oculi: Implications for Variation in Human Facial Expressions,” PLoS One 17, no. 3 (2022): e0264148, 10.1371/journal.pone.0264148. 35231048 PMC8887774

[23] N. G. Isse and M. M. Elahi , “The Corrugator Supercilii Muscle Revisited,” Aesthetic Surgery Journal 21, no. 3 (2001): 209–214, 10.1067/maj.2001.116055. 19331895

[24] H. Rouvière and A. Delmas , Anatomía Humana Descriptiva, Topográfica y Funcional (Editorial Masson, 2005).

[25] C. Muñoz‐Gonzalez and N. Fakih‐Gomez , “Resolving the Controversy Surrounding the Function of the Corrugator Supercilii Muscle,” Aesthetic Plastic Surgery 49, no. 5 (2024): 1444–1457, 10.1007/s00266-024-04454-8. 39448446

[26] R. K. Daniel and B. Landon , “Endoscopic Forehead Lift: Anatomic Basis,” Aesthetic Surgery Journal 17, no. 2 (1997): 97–104, 10.1016/s1090-820x(97)80070-2. 19327696

[27] B. E. Cook, Jr. , M. J. Lucarelli , and B. N. Lemke , “Depressor Supercilii Muscle: Anatomy, Histology, and Cosmetic Implications,” Ophthalmic Plastic and Reconstructive Surgery 17, no. 6 (2001): 404–411, 10.1097/00002341-200111000-00004. 11766019

[28] D. M. Knize , “Muscles That Act on Glabellar Skin: A Closer Look,” Plastic and Reconstructive Surgery 105, no. 1 (2000): 350–361, 10.1097/00006534-200001000-00056. 10627005

[29] A. Domínguez‐Duarte , “Aesthetic Implications of Depressor Supercilii Muscle Block With Botulinum Toxin Type A,” Journal of Cosmetic Dermatology 21, no. 4 (2022): 1374–1378, 10.1111/jocd.14856. 35175677

[30] J. Tong , M. J. Lopez , A. O. Fakoya , and B. C. Patel , “Anatomy, Head and Neck: Orbicularis Oculi Muscle,” in StatPearls [Internet] (StatPearls Publishing, 2024). 28722936

[31] P. Ekman , Emotion in the Human Face (Malor Books, 2015), 456. Originally published 1972.

[32] V. Van Edwards , Cues: Master the Secret Language of Charismatic Communication (Penguin Business, 2022).

[33] V. Van Edwards , Captivate: The Science of Succeeding With People (Portfolio/Penguin, 2017).

[34] D. J. Rams , M. Koziej , J. B. Green , et al., “The Relationship Between Glabellar Contraction Patterns and Glabellar Muscle Anatomy: A Magnetic Resonance Imaging‐Based Study,” Aesthetic Surgery Journal 45, no. 1 (2024): sjae202, 10.1093/asj/sjae202. 39351911

